# Safety and efficacy of low-dose initial citrate infusion for continuous kidney replacement therapy in critically ill children

**DOI:** 10.1007/s00467-026-07234-5

**Published:** 2026-03-10

**Authors:** Muhterem Duyu, Ayşe Aşık

**Affiliations:** Department of Pediatric Intensive Care, Goztepe Prof. Dr. Suleyman Yalcin City Hospital, Istanbul, Turkey 34722

**Keywords:** Circuit survival, Continuous kidney replacement therapy, Regional citrate anticoagulation, Citrate-related complications, Pediatric intensive care

## Abstract

**Background:**

Circuit survival (CS) is critical for the maintenance of continuous kidney replacement therapy (CKRT). Regional citrate anticoagulation (RCA) is widely used, effective, and relatively safe; however, it is associated with complications such as citrate accumulation (CA). We aimed to analyze how a lower citrate infusion dose (CID) protocol could impact CS and risks for CA.

**Methods:**

This retrospective single-center cohort study compared the efficacy and safety of standard CID (3.0 mmol/L) and low CID (2.2–2.5 mmol/L) at initial administration in patients receiving RCA for CKRT in a pediatric intensive care unit.

**Results:**

A total of 127 patients received 239 circuits (115 circuits in 55 recipients of standard CID, and 124 filters in 72 recipients of low CID). When filter life was limited to 72 h, median CS for all filters was similar between the standard and low CID groups (51.0 h [IQR: 25.0–72.0] vs. 49.5 h [IQR: 24.0–72.0]; *p* = 0.857). CS was also similar in circuits terminated due to clotting (standard CID: 38.0 h [IQR: 20.0–58.0], low CID: 37.5 h [IQR: 18.0–55.0]). Recipients of the low CID protocol had significantly lower frequencies of metabolic alkalosis, CA, and hypocalcemia. Multivariate regression identified two independent CA risk factors: longer circuit runtime (OR 1.013, 95% CI 1.002–1.025) and higher weight-adjusted blood flow rates (OR 1.208, 95% CI 1.022–1.427).

**Conclusions:**

Administering a low CID when initializing RCA significantly reduces the likelihood of citrate-related complications while maintaining anticoagulant efficacy.

**Graphical Abstract:**

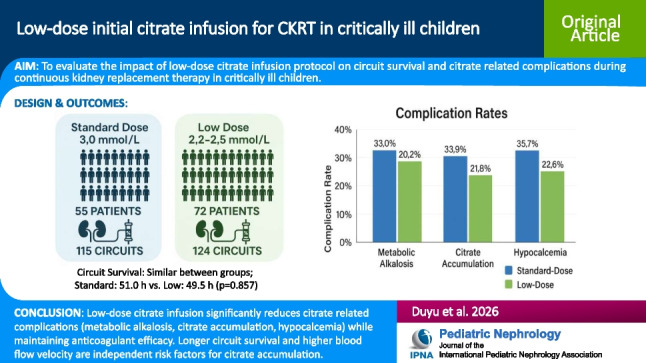

**Supplementary Information:**

The online version contains supplementary material available at 10.1007/s00467-026-07234-5.

## Introduction

Continuous kidney replacement therapy (CKRT), a rather common treatment in the pediatric intensive care unit (PICU), is utilized in the management of various diseases and conditions, including acute kidney injury (AKI), intoxication, acute liver failure, and congenital metabolic diseases [1]. The efficacy of this treatment is associated with safe and reliable maintenance of the intervention, which is dependent on many factors—including circuit survival (CS). Early clotting of CKRT filters reduces therapy efficacy due to downtime and may cause blood loss leading to higher transfusion requirements, hemodynamic instability during circuit reconnection, inadequate solute and fluid control, higher costs, and increased workload for intensive care staff [2, 3].

The most frequently used methods for CKRT are heparin anticoagulation, which has a systemic effect, and citrate anticoagulation, which is accepted to exert a local effect. Particularly in critical illnesses that predispose to bleeding (shock, surgery, trauma, severe liver failure), regional citrate anticoagulation (RCA) significantly reduces the risk of bleeding during CKRT compared to heparin-based anticoagulation [4, 5]. In addition, RCA has been shown to prolong CS relative to heparin, which has caused broader adoption of this anticoagulation method [6–11].

Despite the advantages offered by RCA, it also carries an increased risk of metabolic and electrolyte disorders, including metabolic alkalosis, hypocalcemia, and most critically, citrate accumulation (CA) [5, 8, 12]. It is also crucial to note that there is no consensus on initial citrate infusion dose (CID) protocols in either pediatric or adult CKRT settings, with administered doses ranging from 2 mmol/L to 5 mmol/L [4, 11, 13–16]. Especially in young children, who have reduced hepatic citrate clearance capacity and are at higher risk for CA, a strategy involving the administration of a low initial CID may reduce RCA-related complications without significantly shortening CS [4]. Furthermore, there have been only a few studies that have described the effects of different initial CIDs on CS or other RCA-related complications, and these have been performed among adult CKRT recipients [17–19].

Our primary objective in this study was to evaluate and compare standard initial CID (3.0 mmol/L) and low CID (2.5 and 2.2 mmol/L) in terms of their impacts on CS and to analyze the efficacy of low initial CID in reducing metabolic and electrolyte complications. Our secondary objective was to analyze the risk factors associated with CA, the most feared complication associated with RCA.

## Materials and methods

We performed a retrospective cohort study at our multidisciplinary PICU in Prof. Dr. Suleyman Yalcin City Hospital, a tertiary care center in Istanbul, Turkey. The research plan was approved by the institutional review board (Registration number: 2025/0129). The board waived the need for informed consent since this was a retrospective review.

### Study population

We screened all critically ill children admitted to our general PICU between March 2017 and March 2025 who received CKRT with RCA. Since only heparin anticoagulation had been used in our center prior to March 2017, patients who received CKRT before this date were not included. We excluded patients if they had incomplete records, needed therapeutic anticoagulation or thrombolytic agents either during CKRT or in the 24 h before starting it, or if they got heparin instead of citrate for CKRT.

At our center, the initial CID prescribed for pediatric patients undergoing CKRT between March 2017 and December 2021 was 3.0 mmol/L. After December 2021, a lower initial CID was used for all patients undergoing RCA. According to this protocol, the CID was set at 2.2 mmol/L for patients weighing ≤ 10 kg and 2.5 mmol/L for patients weighing > 10 kg. Thus, based on this division, patients were divided into two groups: those with an initial CID of 3.0 mmol/L (Standard CID) and those receiving 2.2 or 2.5 mmol/L (Low CID) (Fig. S1).

### Patient data

We collected demographic information, laboratory findings, and CKRT-related parameters from electronic health records and physical documentation. At PICU admission, we recorded each patient’s age, weight, sex, other medical conditions, primary diagnosis, and PRISM III severity score. We grouped primary diagnoses at admission into nine categories: renal disorders, hematologic–oncologic conditions, sepsis, pneumonia or respiratory failure, metabolic disease, cardiac arrest, intoxications, cardiac diseases, and others.

We grouped the reasons for starting CKRT into these categories: electrolyte or acid–base imbalance, fluid overload (FO), AKI, hyperammonemia, acute metabolic decompensation, rhabdomyolysis, and tumor lysis syndrome. We identified why CKRT was started by reviewing the attending physician’s notes. We classified patients as survivors if they were discharged alive from the PICU.

Prior to starting CKRT, we collected the following laboratory data: arterial blood gases (pH/lactate), creatinine, urea, serum electrolytes (potassium, sodium, phosphate, magnesium, total calcium, and ionized calcium).

### Administration of CKRT and citrate anticoagulation

We ran all CKRT treatments using Prismaflex machines (models HF20, M60, and M100; Gambro, Sweden/Baxter). Every patient received continuous venovenous hemodiafiltration. We placed double-lumen dialysis catheters sized between 7 and 12 French, choosing the size based on each child’s age and weight.

The standard CID and low CID anticoagulation protocols, including starting parameters, equipment specifications, treatment monitoring procedures, and dose adjustment algorithms, are detailed in the Online Resource (Table S1–S5).During each CKRT circuit, citrate infusion was dynamically adjusted according to a predefined protocol based on simultaneous post-filter and systemic ionized calcium (iCa^++^) measurements, initially performed at 30-min intervals and subsequently at gradually extended intervals up to every 4 h (Table S3–S5). In brief, we tracked these treatment details: how long CKRT ran overall, how many filters we used and how long they lasted, filter and catheter sizes, CS time (both overall and when clotting caused failure), machine settings like blood pump flow and fluid rates, and dose of citrate. We grouped circuit termination reasons into five types: clotting in the circuit, vascular access malfunction, planned 72-h filter changes, end of CKRT treatments (achievement of treatment goals or patient death), and machine alarms or technical glitches. We defined a circuit as clotted if the transmembrane pressure stayed above 250 mmHg or if we could detect any visible clots in the circuit. We identified catheter malfunction when access pressures remained persistently abnormal either excessively negative (below –250 mmHg) or excessively positive (above + 300 mmHg) indicating mechanical catheter obstruction.

### Definitions

CS was measured from CKRT initiation with a given filter until filter removal or session termination. CKRT hemofilter manufacturers do not recommend filter use for more than 72 h. However, because this limit has been exceeded in previous studies and no major complications have been reported, this threshold was not strictly applied in our practice [20–22]. Some circuits were used for longer than 72 h depending on clinical assessments and in cases where it was predicted that CKRT requirements would soon be resolved. Nonetheless, in cases where circuit use exceeded 72 h, CS and RCA-related complication analyses were based on the first 72 h of circuit life.

### Outcomes and statistical analysis

Since citrate undergoes rapid metabolism and its anticoagulant action remains confined to the extracorporeal circuit, we analyzed each circuit as an independent unit rather than clustering by patient. This exploratory analysis used the data of all patients and filters according to the initial CID. Therefore, patient-level analyses (demographics, clinical characteristics) included 127 patients, whereas filter-level analyses (CS, citrate-related metabolic and electrolyte complications) covered all 239 CKRT circuits. When using the Prismaflex device, the filter is integrated into the circuit, making filter life and circuit life synonymous. Continuous variables are presented as median (interquartile range [IQR]) and categorical variables as numbers, proportions, and percentages. For comparing groups, we used chi-square or Fisher’s exact test for categorical data and Mann–Whitney *U* tests for continuous variables since our data did not follow normal distributions. We used logistic regression to examine how CS related to CA, reporting odds ratios with 95% confidence intervals. Risk factors for CA were identified using univariate analysis, and variables with *p* < 0.05 were subsequently entered into a multivariate logistic regression model. Variables with a *p* value of < 0.05 in univariate comparison were included in the multivariate model. We created Kaplan–Meier curves to show CS over time, calculated median survival with confidence intervals for each group, and compared the curves using log-rank tests. To measure how anticoagulation affected clotting risk while accounting for other factors that might matter, such as filter size, blood flow rate, whether we used a femoral catheter, citrate dose, and patient weight, we ran a Cox proportional hazards analysis [4, 17, 23, 24]. We considered results statistically significant at *p* < 0.05. All analyses were done using SPSS version 23.0 (IBM, Chicago, IL). Figures were created with Microsoft Word and IBM SPSS Statistics 23.0.

## Results

Over eight years, 2,376 children came to our PICU, and 136 of them needed CKRT. We started by reviewing all 136 cases, then excluded nine who did not meet our criteria, leaving 127 patients in the final analysis (see Figure S1 for the flow diagram).

### Description of the study population

Among the 127 patients, 55 received standard citrate dosing (3.0 mmol/L) and 72 received lower doses (2.2–2.5 mmol/L). Baseline characteristics were comparable between groups (Table 1), except for catheter insertion site. The low-dose group more often had internal jugular catheters (79.2% vs. 52.7%, *p* = 0.002), while the standard-dose group used femoral catheters more frequently (34.5% vs. 15.3%, *p* = 0.020). The length of PICU stay and PICU survival was similar among the groups.

**Table 1:** Overall summary and comparison of patients receiving standard versus low initial citrate dosage for regional citrate anticoagulation

Variable	All patients (*n* = 127)	Standard CID (*n* = 55)	Low CID (*n* = 72)	*p*
Age (yr), median (IQR)	6.3 (2.1–13.3)	5.2 (1.3–11.3)	6.55 (2.45–13.72)	0.176
Male sex, *n* (%)	60 (47.2)	28 (50.9)	32 (44.4)	0.47
Weight (kg), median (IQR)	21 (11.5–44)	21 (11–42)	22 (12.25–50)	0.452
Weight < 10 kg, *n* (%)	18 (14.2)	9 (16.4)	9 (12.5)	0.717
PRISM III score, median (IQR)	14 (9–21)	14 (10–24)	14 (8–20.75)	0.308
Comorbidity, *n* (%)	59 (46.5)	30 (54.5)	29 (40.3)	0.110
CKRT indication				
Acute kidney injury	52 (40.9)	21 (38.2)	31 (43.1)	0.58
Electrolyte/acid base disturbance	35 (27.6)	19 (34.5)	16 (22.2)	0.180
Fluid overload	22 (17.2)	8 (14.5)	14 (19.4)	0.627
Tumor lysis syndrome	7 (5.5)	4 (7.3)	3 (4.2)	0.465
Rhabdomyolysis	7 (5.5)	1 (1.8)	6 (8.3)	0.138
Hyperammonemia	3 (2.4)	1 (1.8)	2 (2.8)	0.724
Acute attack of metabolic disease	1 (0.8)	1 (1.8)	0	0.251
Insertion site of access catheters				
Internal jugular	86 (67.7)	29 (52.7)	57 (79.2)	0.002*
Subclavian	11 (8.7)	7 (12.7)	4 (5.6)	0.155
Femoral	30 (23.6)	19 (34.5)	11 (15.3)	0.020*
Laboratory variables at initiation of CKRT, median (IQR)				
Creatinine (mg/dL)	2.76 (1.1–4.7)	2.1 (0.9–3.58)	3.35 (1.33–5.3)	0.054
Urea (mg/dL)	102 (51–150)	101 (47–146)	103.5 (51–162)	0.599
pH	7.2 (7.1–7.28)	7.17 (7.08–7.3)	7.21 (7.1–7.26)	0.439
Lactate	2.9 (2.2–5.18)	4 (1.6–9)	2.8 (2.4–3.86)	0.315
Potassium, (mmol/L)	4.2 (3.5–5.1)	4.3 (3.8–5)	3.95 (3.42–5.17)	0.245
Sodium, (mmol/L)	137 (133–141)	137 (133–141)	135.5 (132.1–140)	0.407
Phosphor, (mg/dl)	5.7 (3.4–7)	4.8 (3.4–6.3)	5.9 (3.2–7.2)	0.272
Magnesium, (mg/dl)	2 (1.75–2.45)	2 (1.7–2.3)	1.9 (1.8–2.8)	0.726
Total calcium, (mg/dL)	7.9 (7.2–9.2)	8 (7.4–8.6)	7.8 (6.9–9.9)	0.735
Ionized calcium, (mmol/L)	1.04 (0.98–1.1)	1.03 (0.98–1.1)	1.05 (0.95–1.1)	0.621
Length of PICU stay (days), median (IQR)	8 (5–15)	8 (4–14)	8 (5–17)	0.354
PICU mortality, *n* (%)	33 (26)	19 (34.5)	14 (19.4)	0.086

*RCA*, regional citrate anticoagulation; *CKRT*, Continuous kidney replacement therapy; *PICU*, Pediatric intensive care unit; *PRISM*, Pediatric Risk of Mortality; *IQR*, Interquartile range

^*^p < 0.05, statistically significant

### CKRT and hemofilter characteristics

A total of 12,071 h of dialysis therapy (standard CID: 5,762 h, low CID: 6,309 h) were included in the analysis (Table 2). Median CKRT times per patient were similar between groups (70 h vs. 58.5 h, *p* = 0.24). Across all treatments, we used 239 filters in total: 115 in the standard-dose group and 124 in the low-dose group. Among all circuits, regardless of whether termination was due to clotting or other causes, median CS showed no significant difference: 51.0 h in the standard-dose group versus 49.5 h in the low-dose group (*p* = 0.857). No significant difference was found in the CS of filters whose circuit life expired due to clotting (respectively, median 38.0 h vs. 37.5 h, *p* = 0.301). The distribution of hemofilter and hemodialysis catheter sizes used and the dialysis prescriptions (pump flow rate, dialysate flow rate, replacement flow rate) were also similar in both groups. As expected, the citrate dose was significantly higher in the standard CID group (4 vs. 2.5 mmol/L, *p* < 0.001) (Table 2).

**Table 2:** Characteristics of CKRT in children treated with standard and low CID

Variable	All patients (*n* = 127)	Standard CID (*n* = 55)	Low CID (*n* = 72)	*p*
Total CKRT time (h)	12,071	5762	6309	
CKRT time (h) per patient, median (IQR)	68.0 (34.0–128.0)	70.0 (40.0–144.0)	58.5 (30.2–120.0)	0.240
Total number of hemofilters	239	115	124	
Median CS (h), median (IQR)	51.0 (24.0–70.0)	51.0 (24.0–67.0)	49.5 (26.5–70.0)	0.857
Median CS for clotting hemofilter (h)	38.0 (32.0–55.0)	38.0 (24.0–50.0)	37.5 (32.5–59.0)	0.301
Filter size (m^2^), *n* (%)				
0.2	24 (18.9)	15 (27.3)	9 (12.5)	0.060
0.6	47 (37)	18 (32.7)	29 (40.3)	0.492
0.9	56 (44.1)	22 (40)	34 (47.1)	0.417
Pump flow rate (mL/min), median (IQR)	90 (60–120)	90 (55–110)	96 (60–120)	0.445
< 100, *n* (%)	69 (54.3)	32 (58.2)	37 (51.4)	0.446
≥ 100, *n* (%)	58 (45.7)	22 (41.8)	35 (48.2)	
Dialysate flow rate, (mL/min), median (IQR)	700 (400–1000)	800 (500–900)	600 (360–1170)	0.718
Dialysate flow rate per kg, (mL/kg), median (IQR)	31.8 (20–42.24)	33.4 (23.54–42.8)	27.96 (19.2–42.18)	0.105
Replacement flow rate, (mL/min), median (IQR)	500 (300–800)	500 (300–800)	500 (300–900)	0.992
Replacement flow rate per kg, (mL/min), median (IQR)	21.8 (17.2–33.2)	24 (19.1–29.6)	20 (15–33.2)	0.198
Citrate dose (mmol/L), median (IQR)	2.8 (2.5–4)	4 (3.5–4)	2.5 (2.4–2.7)	< 0.001*
Dialysis catheter size (French), *n* (%)				
7	8 (6.3)	5 (9.1)	3 (4.2)	0.258
8	27 (21.3)	16 (29.1)	11 (15.3)	0.096
9	23 (18.1)	9 (16.4)	14 (19.4)	0.83
10	26 (20.5)	10 (18.2)	16 (22.2)	0.576
11	29 (22.8)	12 (21.8)	17 (23.6)	0.811
12	14 (11)	3 (5.5)	11 (15.3)	0.143

*CKRT*, continuous kidney replacement therapy; *IQR*, interquartile range; *CS*, circuit survival

^*^p < 0.05, statistically significant

While adjustments to citrate dosage were frequently necessary in both cohorts to maintain target post-filter iCa^++^ levels, the initial, median, and final CID remained significantly lower in the low CID group throughout CKRT (initial CID: 2.5 vs. 3.0 mmol/L; median CID: 2.6 vs. 3.8 mmol/L; final CID: 3.0 vs. 4.0 mmol/L; all *p* < 0.001). This finding indicates that the low-dose group did not progressively approach the standard-dose levels (Table S6).

In the standard CID group, there were 11 (9.6%) events of hemofilter removal due to clotting. The low CID group presented with 20 (16.1%) such events, which was similar between both groups (*p* = 0.188). Other causes of circuit termination showed similar distributions between groups (Table 3).

**Table 3:** Reasons for hemofilter disconnection/failure, compared between standard and low citrate dosage

Causes, *n* (%)	All circuits (*n* = 239)	Standard CID (*n* = 115 circuits)	Low CID (*n* = 124 circuits)	*p*
Clotting	31 (13)	11 (9.6)	20 (16.1)	0.188
Vascular access malfunction	27 (11.3)	12 (10.4)	15 (12.1)	0.841
Scheduled filter replacement after 72 h	65 (27.2)	38 (33)	27 (21.8)	0.060
End of CKRT treatment	106 (44.4)	51 (44.3)	55 (44.4)	0.990
Technical issues/alarms	10 (4.2)	3 (2.6)	7 (5.6)	0.502

*RCA*, regional citrate anticoagulation; *CKRT*, continuous kidney replacement therapy

**Fig. 1:**
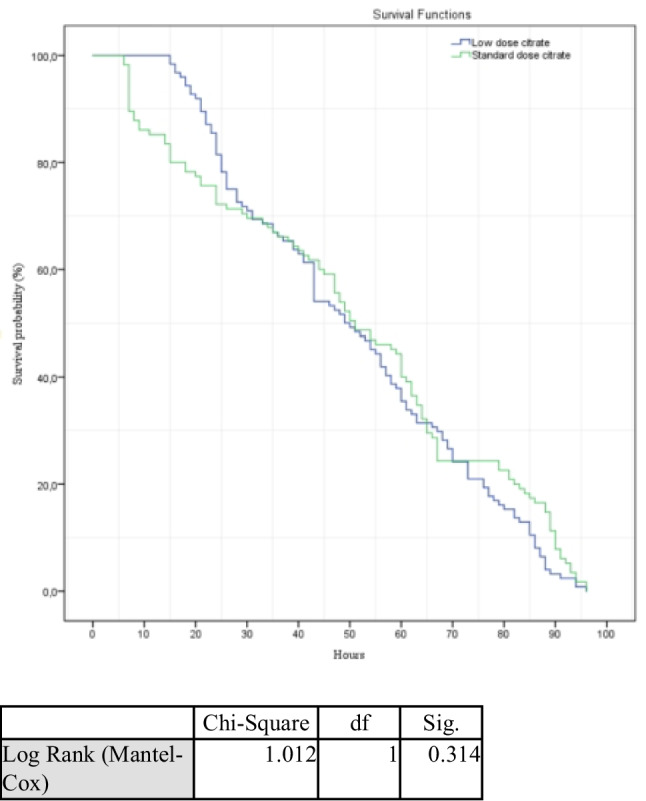
Kaplan–Meier survival curve for overall circuit survival with anticoagulation using standard and low-dose citrate anticoagulation

**Fig. 2:**
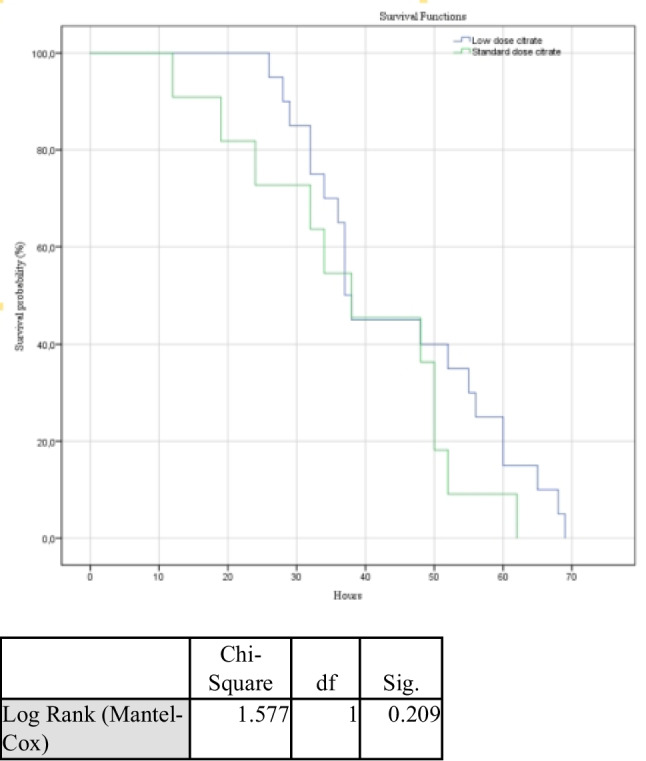
Kaplan–Meier survival curve for overall circuit survival in subgroup analysis based on filter clotting

The log-rank test demonstrated that results were similar in the low-dose and standard-dose CID groups (log-rank *p* = 0.314) (Fig. 1). Similarly, CS time analysis based on clotting of hemofilters was also similar in both groups (log-rank *p* = 0.209) (Fig. 2).

The standard CID group received significantly higher citrate infusion doses at baseline, during CKRT, and at treatment completion compared with the low CID group (all p < 0.001) (Table S6). Cox’s proportional hazard analysis showed that the use of low CID did not significantly increase the risk of hemofilter clotting (median CS time limited by hemofilter clotting) compared to the use of standard CID (HR 1.85; 95% CI 0.55–6.17, *p* = 0.125). Dialysis catheter placement, low weight, small filter size, and low pump flow rates showed no association with hemofilter clotting (Table S7).

### Citrate-related metabolic and electrolyte complications

The most frequent electrolyte and metabolic disturbances were hypocalcemia (35.7% in standard CID, 22.6% in low CID), metabolic alkalosis (33.0% in standard CID, 20.2% in low CID), and hypophosphatemia (30.4% in standard CID, 27.6% in low CID). Among these disturbances, hypocalcemia and metabolic alkalosis were significantly more frequent in the standard CID group than in the low CID group (*p* = 0.032 and *p* = 0.028, respectively). The groups showed similar rates of other electrolyte abnormalities, including hypercalcemia, hyponatremia, hypernatremia, and hypomagnesemia. CA was significantly more frequent in recipients of standard citrate dosage (33.9% vs. 21.8%, *p* = 0.036). Although citrate lock was more common in the standard CID group, statistical significance was not reached (3.5% vs. 0.8%) (Table 4).

**Table 4:** Metabolic and electrolyte disturbances according to initial citrate infusion dose group

Variables, *n* (%)	All circuits (*n* = 239)	Standard CID (*n* = 115 circuits)	Low CID (*n* = 124 circuits)		*p*
Hypocalcemia	69 (28.9)	41 (35.7)	28 (22.6)		0.032*
Hypercalcemia	11 (4.6)	3 (2.6)	8 (6.5)		0.210
Hyponatremia	1 (0.4)	1 (0.8)	0		1.000
Hypernatremia	31 (13)	18 (15.7)	13 (10.5)		0.253
Hypomagnesemia	23 (9.6)	13 (11.3)	10 (8.1)		0.511
Hypophosphatemia	66 (26.6)	35 (30.4)	31 (27.6)		0.386
Metabolic alkalosis	63 (26.4)	38 (33.0)	25 (20.2)		0.028*
CA	66 (27.6)	39 (33.9)	27 (21.8)	0.036*	
Citrate lock	5 (2.1)	4 (3.5)	1 (0.8)	0.198	

*CID*, citrate infusion dose; *CA*, citrate accumulation

^*^p < 0.05, statistically significant

### Citrate accumulation-related risk factors

Bivariate analysis revealed that age, body weight, CS, initial lactate level, standard initial CID group (≥ 3 mmol/L), and weight-adjusted blood flow rate were significantly associated with CA (Table 5).

**Table 5:** Comparison of variables between patients with and without citrate accumulation

Variables	Citrate accumulation		*p*
	No (*n* = 173)	Yes (*n* = 66)	
Age (yr), median (IQR)	9.8 (2.8–14.0)	5.6 (1.3–11.3)	0.024*
Weight (kg), median (IQR)	28.0 (12.5–50.0)	20.0 (10.0–38.5)	0.033*
CS (h), median (IQR)	48.0 (24.0–67.0)	54.5 (35.5–68.5)	0.027*
Initial lactate level (mmol/L), median (IQR)	2.9 (2.2–4.2)	3.6 (2.5–6.0)	0.011*
Initial CID (mmol/L), median (IQR)	2.5 (2.5–3.0)	3.0 (2.5–3.0)	0.090
Average CID (mmol/L), median (IQR)	2.5 (2.5–4.0)	3.5 (2.5–4.0)	0.058
Standard CID dose group (≥ 3 mmol/L), *n* (%)	76.0 (43.9)	39.0 (59.1)	0.043*
Low CID dose group (≤ 2.5 mmol/L), *n* (%)	11.0 (6.4)	7.0 (10.6)	0.279
Blood flow rate (mL/kg/min), median (IQR)	3.7 (2.5–5.0)	4.2 (2.9–5.5)	0.031*
Dialysate flow rate (mL/kg/h), median (IQR)	30.7 (20.0–42.0)	31.5 (23.0–44.7)	0.457
Replacement flow rate (mL/kg/h), median (IQR)	21.0 (16.6–31.7)	21.0 (18.8–28.2)	0.684

*CID*, citrate infusion dose; *CS*, circuit survival

^*^p < 0.05, statistically significant

Parameters with significant relationships were included in the logistic regression model. Univariable logistic regression showed that longer CS (OR: 1.012, 95% CI: 1.001–1.023), higher initial lactate level (OR: 1.076, 95% CI: 1.008–1.148), receiving standard initial CID group (≥ 3 mmol/L) (OR: 1.844, 95% CI: 1.037–3.277), and higher blood flow rate per kg (OR: 1.221, 95% CI: 1.042–1.431) were associated with increased risk of CA. In multivariable analyses, each one-hour increase of the CS was associated with 1.3% greater odds of CA (OR: 1.013, 95% CI: 1.002–1.025). In addition, each mL of increase in blood flow rate (per kg) was found to be associated with 20.0% greater odds of CA (OR: 1.208, 95% CI: 1.022–1.427) (Table 6).

**Table 6:** Citrate accumulation and odds ratios (95% CI) of explanatory factors

Variables	Univariable analysis			Multivariable analysis		
	OR	95% CI	*p*	OR	95% CI	*p*
Age (yr)	0.953	0.907–1.003	0.064			
Weight (kg)	0.985	0.970–1.000	0.052			
CS (h)	1.012	1.001–1.023	0.034*	1.013	1.002–1.025	0.025*
Initial lactate level (mmol/L)	1.076	1.008–1.148	0.027*	1.060	0.988–1.137	0.106
CID group (≥ 3 mmol/L)	1.844	1.037–3.277	0.037*	1.742	0.954–3.183	0.071*
Blood flow rate (mL/kg/min)	1.221	1.042–1.431	0.013*	1.208	1.022–1.427	0.027*

*CS*, circuit survival; *CID*, citrate infusion dose; *CI*, confidence interval; *OR*, odds ratio

^*^p < 0.05, statistically significant

## Discussion

The present study included the analysis of 239 hemofilters administered to 127 patients undergoing CKRT with RCA. Critically, our findings indicate that administering a low CID for the initial RCA protocol offers similar CS to the standard-dose protocol, and that the low CID protocol reduces citrate-related metabolic and electrolyte disturbances (such as hypocalcemia, CA, and metabolic alkalosis). Furthermore, we report that longer CS and higher blood flow rate (per kg) were independent risk factors associated with CA.

Pediatric CKRT uses smaller diameter catheters and hemofilters with lower surface area, and lower blood flow rates are prescribed compared to adults. This puts pediatric patients at a disadvantage in terms of circuit longevity [24]. Therefore, effective anticoagulation is crucial for the uninterrupted maintenance of CKRT in pediatric patients [24, 25]. While heparin anticoagulation was previously the most commonly used anticoagulation method in CKRT patients due to its proven efficacy, RCA has become more prevalent in recent years owing to its limited systemic impact which greatly reduces bleeding risks. Indeed, adult and pediatric studies have shown that RCA prevents bleeding-related complications, reduces transfusion requirements, and prolongs CS, even when compared to heparin anticoagulation [4, 5, 8–10, 12, 14, 22, 24]. However, the breadth of research on this topic has not resulted in a widely accepted consensus regarding the optimal initial CID to prolong CS – in either pediatric or adult literature.

 Despite its advantages, RCA may cause electrolyte and metabolic side effects such as CA, metabolic alkalosis, metabolic acidosis, hypernatremia, and severe hypocalcemia, which can potentially lead to cardiac arrest [26–28]. The heterogeneity among variables used in CKRT studies (age groups, filter selection and replacement criteria, citrate dose and solution composition, CKRT prescriptions, and modalities) leads to significant limitations in analyzing the effects of RCA on circuit life and RCA-related complications [11, 16, 18]. Furthermore, in addition to circuit clotting, causes of circuit failure may also include vascular access malfunctions, cessation of CKRT due to resolving of indication, or technical issues. Therefore, for a more accurate assessment of anticoagulation efficacy, it would be more appropriate to analyze clotting-related circuit failures separately. In our study, in accordance with the literature, detailed analyses were performed only on clotted circuits in the CS assessment [4, 29, 30].

CKRT hemofilter manufacturers do not recommend using filters for more than 72 h. However, previous pediatric studies have reported exceeding this timeframe without any major complications. Therefore, our study did not impose a strict restriction, and some circuits were used for more than 72 h [20, 21]. However, in our study, only the first 72 h were considered in the analyses of complications related to CS and RCA. In relation to the fact that many hemofilters are used for longer periods than recommended, there are studies in the literature reporting longer circuit lifespans [31].

Only a few adult CKRT studies have compared the effects of different starting CIDs on CS and complications [17–19]. Post et al. compared starting doses of 2.2 and 3.0 mmol/L in 122 adult patients and reported that the higher dose prolonged CS (39.6 h vs. 22.9 h), but differences in dialysis prescriptions (lower convective flow, lower % filtration fraction, and priming with heparin in the high-dose group) restrict the interpretation of these results [18]. Similarly, a study comparing different concentrations of citrate solutions (23 vs. 18 mmol/L) found no advantage with the higher dose, while Poh et al. reported no difference in CS between the 2.5 and 3.0 mmol/L doses [17, 19].

The literature lacks reliable studies comparing different initial CID protocols in pediatric patients. Therefore, we examined our results relative to studies reporting low-dose RCA (< 3.0 mmol/L). Studies using low initial CID report median CS values between 34.0 and 71.3 h [7, 20, 21]. The most recent pediatric CKRT study using RCA evaluated the use of low initial CID (< 2.7 mmol/L) and found a median CS of 38.5 h (not limited to 72-h CS) [11]. In our study, although we limited our CS assessment to 72 h and used a lower initial CID (2.2–2.5 mmol/L), we found that a similar CS was achieved (median 37.5 h). Another study similar to ours (except for an initial CID of 4.0 mmol/L) limited its CS assessment to 72 h and analyzed only clotting filters. The authors reported a median CS of 41.0 h [32] similar to our data. Taken together, these results suggest that high initial CIDs may not be a crucial parameter in prolonging CS. In addition to low CID use, variables that could shorten CS (filter size, pump flow, femoral vein catheter, weight, etc.) were evaluated in our study using a proportional hazard analysis model. It was shown that low CID use did not pose an additional risk for circuit clotting compared to standard CID use, based on median CS time limited by hemofilter clotting [4, 17, 23, 24].

Our data demonstrate a sustained separation in final citrate doses between the low and standard CID groups, despite dynamic citrate titration throughout CKRT. This indicates that the low-dose strategy did not represent a delayed escalation toward conventional dosing, and that effective anticoagulation was achieved with a lower overall citrate burden without compromising circuit survival. This finding contrasts with previous reports. Poh et al. [17] compared initial CIDs of 2.5 and 3.0 mmol/L in adults, while Kıhtır et al. [11] grouped pediatric circuits by initial CID below and above 2.7 mmol/L-bf. Both studies reported that the differences in citrate dosing between the groups were no longer evident after 24 and 48 h of CKRT, respectively. In our study, however, the designed low-dose protocol, which included a distinct starting dose and dose-adjustment algorithm, successfully maintained a persistent dose separation throughout CKRT. Final CID remained significantly lower in the low CID group (3.0 vs. 4.0 mmol/L), demonstrating that a low-dose strategy can provide a sustainably lower citrate exposure, potentially offering a wider safety margin against citrate-related metabolic complications.

The most feared complication of RCA is CA [7]. Approximately 20–50% of citrate–calcium complexes are removed by the filter, with the remainder metabolized by the Krebs cycle [33]. In addition to conditions such as liver failure, shock, and hypoxemia, neonates and young infants are particularly vulnerable due to relatively immature liver function, which may result in impaired citrate metabolism and subsequent accumulation [34, 35]. If impaired citrate metabolism persists, metabolic acidosis and systemic hypocalcemia can develop, leading to reduced cardiac output and hypotension secondary to myocardial depression. This terminal state is referred to as citrate lock or citrate toxicity [36]. As in our study, a total-to-ionized calcium ratio (T/iCa^++^) greater than 2.5 is generally used as an indirect marker of CA [11, 37]. However, no consensus definition of CA has been validated in either pediatric or adult patients, making recognition and management challenging for clinicians [13]. Some studies define CA solely by the T/iCa^++^ ratio, and report a high incidence, whereas others diagnose fewer cases due to applying multiple criteria that incorporate acid–base status and anion gap [4, 7, 32]. Although no consensus definition exists, the reported incidence of CA in pediatric CKRT studies ranges from 0% to 39.3% [4, 7, 11, 12, 16, 31, 32]; whereas adult studies have reported far lower frequencies (0–2.9%) [37, 38]. The higher risk of CA during RCA in pediatric patients compared with adults can be explained by two factors. First, citrate infusion rates are adjusted according to blood flow rather than body weight. Since blood flow rates (mL/min/kg) are disproportionately higher in small children, citrate must be administered at higher doses to compensate [39]. Second, while citrate clearance is reported as 35–50% in adult CKRT patients, this rate is lower (around 20%) in children [33]. Particularly in small children and neonates, strategies such as using lower citrate concentrations (< 2.5 mmol/L) and maintaining blood flow below 50 mL/min may help reduce the risk of CA [11, 13, 14].

Our study analyzed potential risk factors associated with CA. Each 1-h increase in CS increased the risk by 1.3%. This could be explained by increased citrate exposure due to longer CKRT duration and a decrease in citrate clearance resulting from lower filter efficiency over time [11, 40]. Similarly, Kıhtır et al. reported that an increase in CS resulted in a greater risk of CA [11]. Another independent risk factor for CA identified in our study was blood flow velocity. Accordingly, every 1 mL/min increase in blood flow rate (per kg body weight) increased the risk of CA by 20%. This may be related to the increased citrate load due to greater blood circulation, even without increasing the citrate dose. Given that higher blood flow rates per kg are used, particularly in infants, choosing lower initial CIDs could be a strategy to reduce the risk of CA.

Metabolic alkalosis is another common metabolic complication among recipients of RCA for CKRT. Around 50–80% of citrate entering the systemic circulation is metabolized in the liver into the weak basic compound, bicarbonate. As such, in patients with sufficient metabolic capacity in the liver, excessive citrate exposure might result in alkalosis [4]. Indeed, the prevalence of RCA-associated metabolic alkalosis has been reported to range from 2.0% to 86.5% in pediatric studies [8, 12, 14, 29, 30, 39]. In our study, the frequency of metabolic alkalosis was significantly reduced in patients treated with a low-dose citrate protocol (33.0% vs. 20.2%), a result similar to that described by Kıhtır et al. [11].

Another serious complication associated with RCA is hypocalcemia. In the pediatric literature, the prevalence of hypocalcemia in CKRT patients undergoing RCA ranges from 0% to 60.0% [4, 8, 12, 14, 29, 30, 39]. In our study, the prevalence of hypocalcemia was 28.9%, and low initial CID administration was shown to significantly reduce the risk of hypocalcemia (35.7% vs. 22.6%). In our cohort, all other complications associated with hypocalcemia and citrate were managed conservatively, and none of the patients required switching to alternative anticoagulation.

One of the strengths of our study is its inclusion of a large cohort encompassing data on CKRT in critically ill children over an 8-year period. In addition, our study is the first to compare different initial CIDs in pediatric CKRT patients. The close similarity in the prescription and monitoring algorithms (starting parameters, equipment and solutions, target calcium levels, etc.) used in both protocols (with the exception of the initial CID) allows for a more reliable assessment of the effects of dose differences. Furthermore, the high number of cycles and the long total CKRT durations achieved in the pediatric literature further enhance the scientific strength of the study and provide important implications for clinical practice. However, this was a single-center, retrospective observational study, which is its primary limitation. Therefore, the findings require confirmation in multicenter studies including larger cohorts. Second, the implementation of protocols in two different time periods may have introduced potential biases associated with changes in management and other healthcare practices throughout time. Third, we did not account for circuit pressures or the effect of staff responses to pump alarms, which may be important since ineffective troubleshooting can lead to circuit clotting due to prolonged blood pump interruptions. Finally, our study evaluated survival only during the PICU stay; outcomes such as kidney recovery and long-term mortality were not assessed.

The novel outputs of this study include its comparison of two different initial CID protocols and the examination of their effects on filter life and citrate-related complications. Our findings demonstrate that a lower initial CID (2.2–2.5 mmol/L) significantly reduces the incidence of citrate-related complications, particularly CA and hypocalcemia, while maintaining anticoagulant efficacy. In addition, longer CID and higher blood flow rate (per kg body weight) were identified as independent risk factors for CA. Large-scale studies involving multiple centers that employ prospective designs are needed to optimize citrate dosing strategies and evaluate the factors that increase the risk of CID-related complications.

## Supplementary Information

Below is the link to the electronic supplementary material.

ESM 1 (DOCX.50.0 KB)

## Data Availability

The datasets generated and/or analyzed during the current study are not publicly available due to institutional and ethical restrictions, but are available from the corresponding author on reasonable request.
