# Supplementary material for: Safety and efficacy of low-dose initial citrate infusion for continuous kidney replacement therapy in critically ill children
Source: Pediatr Nephrol. 2026 Mar 10;41(8):2635–45. doi: 10.1007/s00467-026-07234-5 (PMC13337807; doi:10.1007/s00467-026-07234-5)
Supplement: Supplementary file 1 — (DOCX.50.0 KB) [file 467_2026_7234_MOESM1_ESM.docx]

**Figure S1. Flowchart of the study.**

CKRT with low-dose RCA

(Low CID)

- 72 patients
- 124 circuits

CKRT with standard-dose RCA (Standard CID)

- 55 patients
- 115 circuits

Patients who received continuous kidney replacement therapy (CKRT) with regional citrate anticoagulation (RCA)

(n = 127)

Excluded patients (n = 9)

- Insufficient data (n = 2)

- Therapeutic anticoagulation treatment (n = 3)

- CKRT with heparin anticoagulation (n = 4)

Patients who received continuous kidney replacement therapy (CKRT)

(n = 136)

2376 critically-ill children admitted to the PICU, from March 2017 to July 2025

PICU: Pediatric intensive care unit, CKRT: continuous kidney replacement therapy, RCA: Regional citrate anticoagulation, CID: Citrate infusion dose

**Regional citrate anticoagulation protocol for critically ill children requiring continuous kidney replacement therapy**

**Starting Parameters**

1. **Blood flow rate:** 3–8 mL/kg/min (maximum 150 mL/min) with respect to patient weight, catheter size, and hemofilter surface [41].

**Regional citrate anticoagulation protocol for critically ill children requiring continuous kidney replacement therapy**

**Starting Parameters**

1. **Blood flow rate:** 3–8 mL/kg/min (maximum 150 mL/min) with respect to patient weight, catheter size and hemofilter surface [40].

**Table S1.** Blood flow rates according to body weight in CKRT patients

| Patient weight (kg) | Blood flow rate (mL/kg/min) |
| --- | --- |
| 3-6 | 8-12 |
| 6-15 | 5-8 |
| 15-30 | 4-6 |
| >30 | 2-4 |

CKRT: continuous kidney replacement therapy

1. **Total effluent dose:** Between 2–3 L/1.73m^2^/h for the majority of patients, while higher total clearance rates (up to a maximum of 5 L/1.73m^2^/h) were reached in cases with specific indications, such as intoxication or hyperammonemia.
2. **Citrate dose:** From March 2017 to December 2021, patients undergoing CKRT with RCA at our center were prescribed an initial citrate infusion dose of 3.0 mmol/L. After December 2021, all RCAs were administered with a low initial citrate infusion dose. Lower dosages were administered as 2.2 mmol/L for patients weighing ≤10 kg and as 2.5 mmol/L for those weighing >10 kg. Citrate flow was coupled to blood flow and adjusted by the CKRT device to achieve the prescribed citrate dose (2.2, 2.5 or 3 mmol/L). All CKRTs receiving RCA were performed with pre-filter citrate anticoagulation and post-filter replacement fluid.
3. **Filtration fraction:** While regulating dialysate and replacement flows, care was taken not to increase the filtration fraction above 25%.
4. **Modality:** Continuous veno-venous hemodiafiltration was administered to all subjects.

**Equipment and solutions**

1. **Device:** Prismaflex system (Gambro-Baxter, Deerfield, IL, USA)
2. **Catheter selection:** Venous access was obtained by dual lumen hemodialysis catheters (7 F to 12 F) depending on the age and weight of the child [40].

**Table S2.** Catheter size selection according to body weight in CKRT patients

| Patient weight | Catheter size |
| --- | --- |
| 3-6 kg | 7 French |
| 6-15 kg | 8-9 French |
| 15-30 kg | 9-10 French |
| >30 kg | 10-12 French |

CKRT: continuous kidney replacement therapy

1. **Circuit selection:** Poly aryl ethylene sulphone (PAES) membranes (Prismaflex HF20; circuit volume 60 mL, surface area 0.2 m^2^) or AN69 membranes (Prismaflex M60; circuit volume 93 mL, surface area 0.6 m^2^ and Prismaflex M100; circuit volume 152 mL, surface area 0.9 m^2^) were used. Children with a body weight of <10 kg underwent CKRT using a Prismaflex HF20 set, those with a body weight between 10 kg and 25 kg received the Prismaflex M60 set, while those exceeding 25 kg received a Prismaflex M100 set.
2. **Circuit priming:** Heparinized saline (5 U/mL) was the primary method used for hemofilter priming. In patients with high risk for hemorrhage however, the circuit was primed via the blood-priming method, particularly among children with a bodyweight of less than 10 kg and those with hemoglobin values below 10 g/dL. For this purpose, erythrocyte suspension was diluted 1-to-1 with saline.
3. **Citrate solution:** Prismocitrate 18/0 (Baxter, USA) (contains 140 mmol/L sodium, 18 mmol/L citrate)
4. **Dialysate solution:** Prism0cal B22 (Baxter, USA) (contains 140 mmol/L sodium, 22 mmol/L HCO_3_^-^)
5. **Replacement solution:** Dialisan (Baxter, USA) (contains 140 mmol/L sodium, 2 mmol/L potassium, 32 mmol/L HCO_3_^-^)
6. **Calcium solution:** Citrate effect was neutralized using a continuous calcium infusion, calcium gluconate 10% 50/50 with dextrose 5% (116 mmol/L). Calcium infusion was administered through a different central venous line. In cases where a separate central line was not available, we administered calcium infusion through the return line of the circuit.

**Treatment Monitoring**

1. **Ionized calcium target:** For both citrate initiation protocols (3.0 mmol/L vs. 2.2-2.5 mmol/L) the post-filter target iCa^++^ level was between 0.25 and 0.35 mmol/L and the patient’s iCa^++^ target was between 1.0 and 1.2 mmol/L. In the standard dose citrate group (3.0 mmol/L), the initial calcium infusion rate was set at 1 mL/kg/h. In the low-dose group (2.2-2.5 mmol/L administered), calcium infusion rate was initiated according to the following formula: citrate flow rate (mL/h) × 0.03. Dose adjustments based on post-filter iCa^++^ and patient iCa^++^ levels were made according to Table S3 among standard dose recipients, and according to Table S4 and Table S5 among the low-dose recipients. Post-filter iCa^++^ and patient iCa^++^ levels were evaluated simultaneously. The monitoring of iCa^++^ concentrations was performed at several time points to ensure maintenance of target concentrations: at the 30th minute, the first, second and fourth hour of therapy, and then routinely every 4 h. Patient calcium levels were also checked one hour after any change in blood flow, citrate concentration, or dialysis.

**Table S3.** Dose adjustment table for the standard citrate dose regimen (3.0 mmol/L)

|  | | Post-filter ionized calcium level (mmol/L) | | | |
| --- | --- | --- | --- | --- | --- |
|  |  | <0.25 | 0.25-0.35 | 0.35-0.6 | >0.6 |
| Patient ionized calcium level (mmol/L) | > 1.2 | Citrate flow rate  **reduce** 0.2 mmol/L | **Maintain** citrate flow rate | Citrate flow rate **increase** 0.2 mmol/L | Citrate flow rate **increase** 0.4 mmol/L |
|  |  | Calcium flow rate  **reduce** 10% | Calcium flow rate **reduce** 10% | Calcium flow rate  **reduce** 10% | Calcium flow rate  **reduce** 10% |
|  | 1  1.0- 1.2 | Citrate flow rate  **reduce** 0.2 mmol/L | **Maintain** citrate flow rate | Citrate flow rate increase 0.2 mmol/L | Citrate flow rate **increase** 0.4 mmol/L |
|  |  | **Maintain** calcium flow rate | **Maintain** calcium flow rate | **Maintain** calcium flow rate | **Maintain** calcium flow rate |
|  | 0.9-1.0 | Citrate flow rate  **reduce** 0.2 mmol/L | **Maintain** citrate flow rate | Citrate flow rate **increase** 0.2 mmol/L | Citrate flow rate **increase** 0.4 mmol/L |
|  |  | Calcium flow rate **increase** 10% | Calcium flow rate **increase** 10% | Calcium flow rate **increase** 10% | Calcium flow rate **increase** 10% |
|  | <0.9 | Citrate flow rate  **reduce** 0.2 mmol/L | **Maintain** citrate flow rate | Citrate flow rate **increase** 0.2 mmol/L | Citrate flow rate **increase** 0.4 mmol/L |
|  |  | Calcium flow rate **increase** 20% | Calcium flow rate **increase** 20% | Calcium flow rate **increase** 20% | Calcium flow rate **increase** 20% |

**Table S4.** Dose adjustment table for low citrate dose regimen (2.2-2.5 mmol/L) in patients weighing ≤ 20 kg

| ≤ 20 kg | | Post-filter ionized calcium level (mmol/L) | | | |
| --- | --- | --- | --- | --- | --- |
|  |  | <0.25 | 0.25-0.35 | 0.35-0.6 | >0.6 |
| Patient ionized calcium level (mmol/L) | >1.2 | Flow rate **reduce**  2 mmol/L | **Maintain** citrate flow rate | Citrate flow rate **increase** 0.2 mmol/L | Citrate flow rate **increase** 0.4 mmol/L |
|  |  | Calcium flow rate **reduce** 2.4 mL/h | Calcium flow rate **reduce**2.4 mL/h | Calcium flow rate  **reduce** 2.4 mL/h | Calcium flow rate  **reduce** 2.4 mL/h |
|  | 1.0-1.2 | Citrate flow rate **reduce** 0.2 mmol/L | **Maintain** citrate flow rate | Citrate flow rate **increase** 0.2 mmol/L | Citrate flow rate **increase** 0.4 mmol/L |
|  |  | **Maintain** calcium flow rate | **Maintain** calcium flow rate | **Maintain** calcium flow rate | **Maintain** calcium flow rate |
|  | 0.9-1.0 | Citrate flow rate  **reduce** 0.2 mmol/L | **Maintain** citrate flow rate | Citrate flow rate **increase** 0.2 mmol/L | Citrate flow rate **increase** 0.4 mmol/L |
|  |  | Calcium flow rate **increase** 2.4 mL/h | Calcium flow rate **increase** 2.4 mL/h | Calcium flow rate **increase** 2.4 mL/h | Calcium flow rate **increase** 2.4 mL/h |
|  | <0.9 | Citrate flow rate  **reduce** 0.2 mmol/L | **Maintain** citrate flow rate | Citrate flow rate **increase** 0.2 mmol/L | Citrate flow rate **increase** 0.4 mmol/L |
|  |  | Calcium flow rate **increase** 4.8 mL/h | Calcium flow rate **increase** 4.8 mL/h | Calcium flow rate **increase** 4.8 mL/h | Calcium flow rate **increase** 4.8 mL/h |

**Table S5.** Dose adjustment table for low citrate dose regimen (2.2-2.5 mmol/L) in patients weighing > 20 kg

| >20 kg | | Post-filter ionized calcium level (mmol/L) | | | |
| --- | --- | --- | --- | --- | --- |
|  |  | <0.25 | 0.25-0.35 | 0.35-0.6 | >0.6 |
| Patient ionized calcium level (mmol/L) | >1.2 | Flow rate **reduce**  2 mmol/L | **Maintain** citrate flow rate | Citrat flow rate **increase** 0.2 mmol/L | Citrat flow rate **increase** 0.4 mmol/L |
|  |  | Calcium flow rate **reduce** 4.8 mL/h | Calcium flow rate **reduce** 4.8 mL/h | Calcium flow rate  **reduce** 4.8mL/h | Calcium flow rate  **reduce 4**.8 mL/h |
|  | 1.0-1.2 | Citrate flow rate **reduce** 0.2 mmol/L | **Maintain** citrate flow rate | Citrat flow rate **increase** 0.2 mmol/L | Citrat flow rate **increase** 0.4 mmol/L |
|  |  | **Maintain** calcium flow rate | **Maintain** calcium flow rate | **Maintain** calcium flow rate | **Maintain** calcium flow rate |
|  | 0.9-1.0 | Citrate flow rate  **reduce** 0.2 mmol/L | **Maintain** citrate flow rate | Citrate flow rate **increase** 0.2 mmol/L | Citrate flow rate **increase** 0.4 mmol/L |
|  |  | Calcium flow rate **increase** 4.8 mL/h | Calcium flow rate **increase** 4.8 mL/h | Calcium flow rate **increase** 4.8 mL/h | Calcium flow rate **increase** 4.8 mL/h |
|  | <0.9 | Citrate flow rate  **reduce** 0.2 mmol/L | **Maintain** citrate flow rate | Citrate flow rate **increase** 0.2 mmol/L | Citrate flow rate **increase** 0.4 mmol/L |
|  |  | Calcium flow rate **increase** 9.6 mL/h | Calcium flow rate **increase 9**.6 mL/h | Calcium flow rate **increase** 9.6 mL/h | Calcium flow rate **increase** 9.6 mL/h |

1. **Assessment of citrate-related metabolic and electrolyte disturbance**

Electrolyte-related and metabolic disturbances during CKRT were recorded and categorized as follows: hypocalcemia (iCa^++^ <1.0 mmol/L), hypercalcemia (iCa^++^ >1.25 mmol/L), hyponatremia (Na <130 mmol/L), hypernatremia (Na >145 mmol/L), hypomagnesemia (<1.5 mg/dL), hypophosphatemia (<2.5 mg/dL), metabolic acidosis (pH <7.35 or base excess [BE] <−3), and metabolic alkalosis (pH >7.45 or BE >+3). Citrate accumulation was defined as a ratio of total-to-ionized calcium ratio (T/iCa^++^) of >2.5 without metabolic acidosis and hypocalcemia [11,38,41]. Citrate lock syndrome was defined as a (T/iCa^++^ of >2.5 with high anion gap metabolic acidosis [37].

**Table S6.** Changes in citrate infusion dose (CID) during CKRT according to anticoagulation strategy

| Variables  (mmol/L, median (IQR)) | Standard CID (n=115 circuits) | Low CID (n=124 circuits) | *p* |
| --- | --- | --- | --- |
| Initial CID | 3.0 (3.0-3.0) | 2.5 (2.2-2.5) | <0.001 |
| Median CID during CKRT | 3.8 (3.4-4.0) | 2.6 (2.4-2.8) | <0.001 |
| Final CID | 4.0 (3.6-4.2) | 3 (2.6-3.2) | <0.001 |

CID: **Citrate infusion** dose; CKRT: Continuous kidney replacement therapy; IQR: Interquartile range

**Table S7.** Cox’s proportional risk analysis of risk of hemofilter clotting

| Variables | Hazard ratio | Standard error | *p* | 95% Confidence interval |
| --- | --- | --- | --- | --- |
| Femoral vein catheter | 1.116 | 0.503 | 0.827 | 0.41-2.98 |
| Weight <10 kg | 1.837 | 0.716 | 0.396 | 0.45-7.46 |
| Filter size 0.2 m² | 2.754 | 0.837 | 0.226 | 0.55-13.72 |
| Pump flow rate <100 mL/min | 1.797 | 0.556 | 0.291 | 0.60-5.33 |
| Citrate dose <3 mmol/L | 1.851 | 0.615 | 0.125 | 0.55-6.17 |
